# Computed tomography measurements of presumptively normal canine sternal lymph nodes

**DOI:** 10.1186/s12917-020-02497-y

**Published:** 2020-08-03

**Authors:** Ladislav Stehlík, Hana Vitulová, Francesco Simeoni, Pavel Proks, Massimo Vignoli

**Affiliations:** 1 Small Animal Clinic, Faculty of Veterinary Medicine, University of Veterinary and Pharmaceutical Sciences Brno, Palackého tř. 1946/1, 612 42 Brno, Czech Republic; 2 Faculty of Veterinary Medicine, University of Teramo, Piano D’Accio, Italy

**Keywords:** dog, thorax, sternal lymph centre, lymph node size, anatomy

## Abstract

**Background:**

There is a lack of information regarding the CT appearance of sternal lymph nodes in dogs. This retrospective anatomic study was aimed to describe the general appearance of sternal lymph nodes in healthy dogs.

**Results:**

Twenty-seven dogs with no abnormality in blood work, urinalysis and CT images were included in the study. Dogs were divided into three weight groups; ≤10 kg, 10.1 to 30 kg and ≥ 30.1 kg. Multi-planar reconstructions of CT images were made to identify sternal lymph nodes. The number, location, size, density and heterogeneity of sternal lymph nodes were recorded. Density and heterogeneity of lymph nodes were measured on pre- and postcontrast images. Except for one dog, sternal lymph nodes were identified in all the dogs. The mean number of sternal lymph nodes per dog was 2.1 (SD 0.6), and the most frequent localisation was at the level of the second sternebra (23 dogs; 85%). There was a positive correlation between the weight and all the dimensions of sternal lymph nodes. A significant negative correlation was found between the age and dorsoventral dimension of the lymph node. Short-to-long axis ratios were not significantly different between the weight groups. None of the measured dimensions nor the ratio values was significantly different between the medium-sized dogs (10.1 to 30 kg) and the large dogs (≥ 30.1 kg). There was a significant difference between precontrast and postcontrast density and heterogeneity values of sternal lymph nodes.

**Conclusions:**

Based on the results, we recommend using the short-to-long axis ratios for sternal lymph node size evaluation among dogs of different size. Sternal lymph nodes in this study appeared on precontrast examination as heterogeneous, and homogenous on the postcontrast examination.

## Background

Sternal lymph nodes (SLN) are the only lymph nodes in the ventral thoracic lymph centre in dogs.[1] Mostly there is one node on each side of the sternum, but some variability in number has been described, and sometimes the lymph nodes are not present.[1–3] They are located dorsal to the sternum, medial to the second costal cartilage and cranioventral to the internal thoracic artery and vein.[1, 3] The lymph to SLN is coming from the sternum, ribs, thymus, serous membranes, adjacent muscles, peritoneal cavity, pelvic cavity and thoracic mammary glands.[1, 3–5] Normal SLN is generally not visible on radiographs, but in large dogs could be seen.[6, 7] Various neoplastic, inflammatory and hematologic diseases affecting the draining area of SLN can change the appearance of SLN.[2, 3, 8–10] Enlargement of SLN can also be found in patients with hemoperitoneum.[10] Only a few studies describing canine SLN on computed tomography (CT) were published.[3, 5, 8].

This study aimed to describe the CT appearance of SLN in a group of healthy dogs and to provide guidelines for clinical practice.

## Results

In one dog, 4-month-old Yorkshire Terrier, the SLN could not be identified on the CT images. This dog was excluded from the statistical analysis, and only 27 dogs were used for the study. There were 17 different breeds represented by Rhodesian Ridgebacks (4; 14.8%), Yorkshire Terrier (3; 11.1%), Labrador Retriever (3; 11.1%), Beagle (2; 7.4%), German Shepherd (2; 7.4%), Poodle (2; 7.4%) and one (3.7%) of each (Australian Shepherd, Bohemian Shepherd, Bloodhound, Crossbreed, Czechoslovakian Wolfdog, Fox terrier, German Spitz, Hungarian Pointing Dog, Shih-Tzu, Staffordshire Bullterrier, West Highland White Terrier). There were 5 (18.5%) intact females, 4 (14.8%) spayed females, 17 (63%) intact males and one (3.7%) castrated male. The mean age of the dogs was 7.4 years (SD 3.08) with a range from 1.3 to 12.2 years. The mean weight was 23.9 kg (SD 16.11), and the range was from 2.6 to 58 kg. Nine dogs (33%) were in category ≤ 10 kg, eight (30%) were in the category between 10.1 and 30 kg and ten dogs (37%) were ≥ 30.1 kg.

**Fig. 1:**
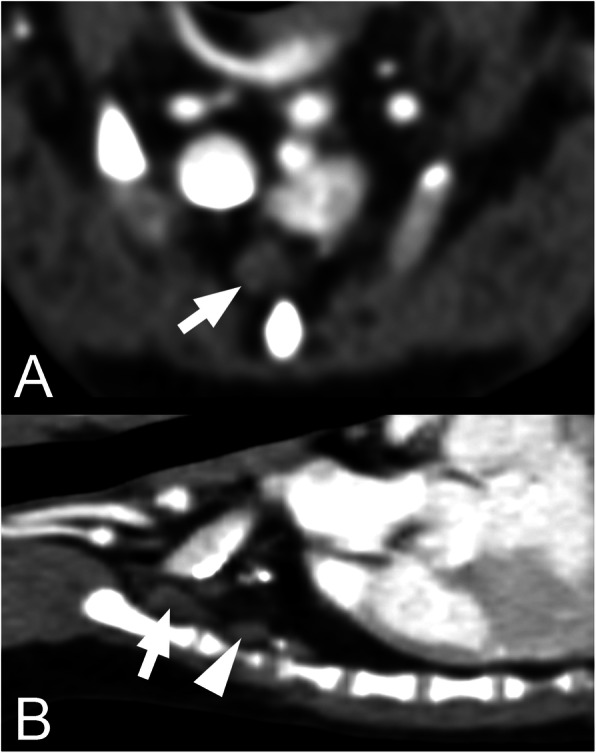
Transversal and sagittal postcontrast CT images with sternal lymph nodes in a small dog .Transversal (**a**) and sagittal (**b**) plane postcontrast CT images of the thorax of a small dog (≤ 10 kg) showing the normal sternal lymph nodes (arrow and arrowhead) and their localisation. The right side of the animal is on the left side of the transversal image

**Fig. 2:**
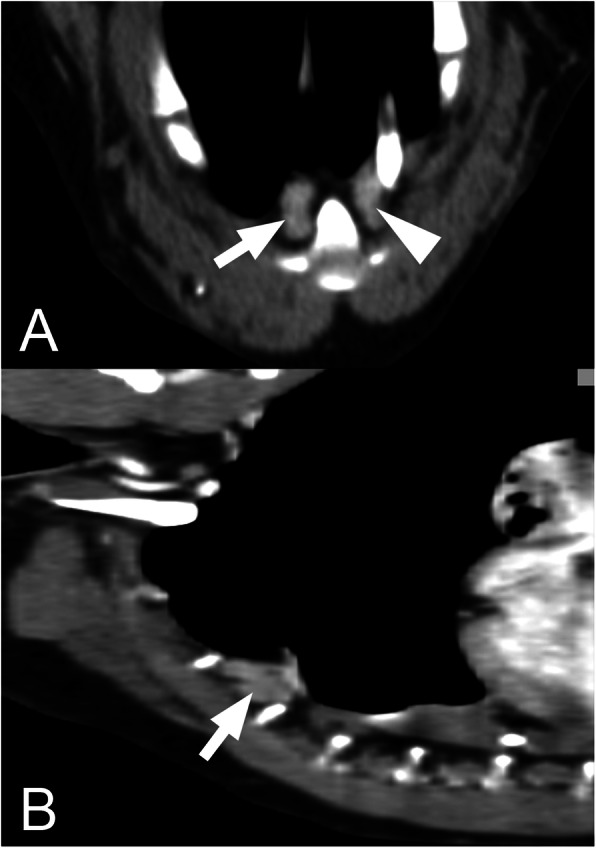
Transversal and sagittal postcontrast CT images with sternal lymph nodes in a medium-size dog. Transversal (**a**) and sagittal (**b**) plane postcontrast CT images of the thorax of a medium-size dog (10.1 to 30 kg) showing the normal sternal lymph nodes (arrow and arrowhead) and their localisation. The right side of the animal is on the left side of the transversal image

**Fig. 3:**
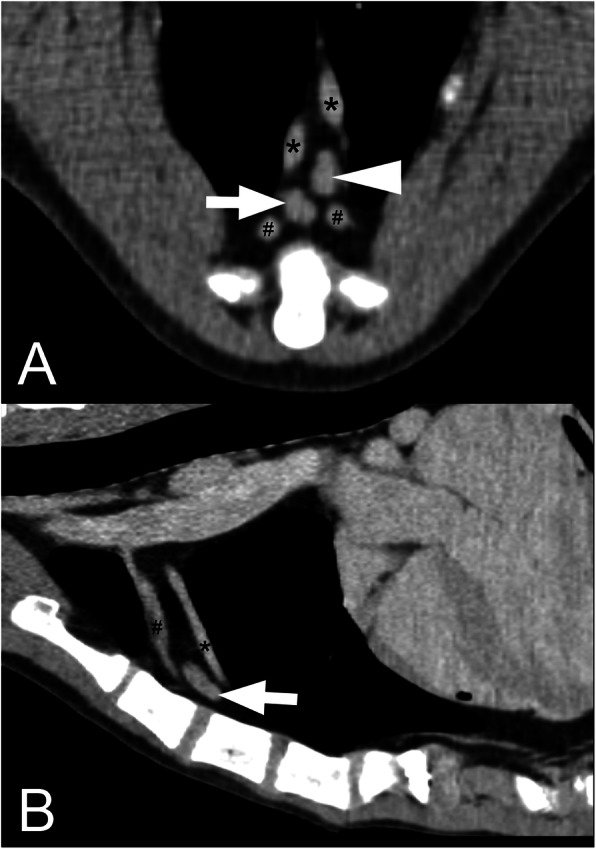
Transversal and sagittal postcontrast CT images with sternal lymph nodes in a large dog. Transversal (**a**) and sagittal (**b**) plane postcontrast CT images of the thorax of a large dog (≥ 30.1 kg) showing the normal sternal lymph nodes (arrow and arrowhead) and their localisation. Internal thoracic artery (*) and vein (#) are also displayed. The right side of the animal is on the left side of the transversal image

The mean number of SLN was 2.1 (SD 0.6). Three dogs (11%) had only one SLN, 17 (63%) dogs had two SLN, and seven (26%) dogs had three SLN. The most frequent localisation of SLN was at the level of the second sternebra (23 dogs; 85%) (Figs. 1, 2 and 3). The exact SLN localisation is shown in Table 1.

**Table 1:** Location of the sternal lymph nodes related to the adjacent sternebrae

Position of SLN	Number of dogs (*n* = 27)	Percentage
1st sternebra	2	7.4
1st − 2nd sternebra	6	22.2
2nd sternebra	7	26.0
2nd − 3rd sternebra	10	37.0
3rd sternebra	2	7.4

Except for one dog, the lymph nodes were well defined with smooth margins (26 dogs, 96.3%). The dog in which the lymph node margins were not smooth and sharp was a Yorkshire Terrier weighing 3 kg, and the SLN dimension was 5.1 × 2.1 × 1.7 mm.

The longest dimension of SLN was in craniocaudal (CrCd) direction, and the shortest was in laterolateral (LL) direction. Descriptive statistics of SLN size is shown in Table 2, and the ratio values are in Table 3.

**Table 2:** Descriptive statistics of the size of the lymph nodes in all three dimensions (*n* = 27)

Statistical variable	CrCd (mm)	DV (mm)	LL (mm)
Mean ± SD	12.4 ± 5.02	5.3 ± 2.30	4.6 ± 1.58
95% CI for the mean	10.46 to 14.43	4.36 to 6.18	3.99 to 5.24
Minimum	2.5	1.3	1.7
Median	11.4	5.2	4.7
Maximum	21.5	11.2	7.1

**Table 3:** Descriptive statistics of the ratio values of the lymph node size (*n* = 27)

	SLN to second sternebra ratio			SLN short-to-long axis ratio	
**Statistical variable**	**CrCd/St2DV**	**DV/St2DV**	**LL/St2DV**	**DV/CrCd**	**LL/CrCd**
Mean ± SD	2.6 ± 1.44	0.5 ± 0.17	0.4 ± 0.17	0.45 ± 0.15	0.42 ± 0.20
95% CI for the mean	2.02 to 3.16	0.40 to 0.54	0.36 to 0.50	0.39 to 0.50	0.34 to 0.50
Minimum	1.3	0.2	0.2	0.11	0.16
Median	2.4	0.4	0.4	0.41	0.36
Maximum	9.1	1.0	1.0	0.75	1.24

*SLN *sternal lymph node; *St2DV *dorso-ventral dimension of the 2nd sternebra

There was positive correlation between the weight and the CrCd (*r* = 0.584, *p* = 0.0014), DV (*r* = 0.503, *p* = 0.0075), LL (*r* = 0.639, *p* = 0.0003) dimensions of SLN and also St2DV dimension (*r* = 0.891, *p* < 0.001). From all the ratio values, only LL/St2DV was found to correlate with the weight (*r*=-0.441, *p* = 0.02). In the case of age, there was only one significant correlation with the SLN DV dimension (*r*=-0.528, *p* = 0.0046). All the other parameters (SLN CrCd, SLN LL, St2DV, CrCd/St2DV, DV/St2DV, LL/St2DV, DV/CrCd, LL/CrCd) were not significantly correlated with the age (*p* > 0.05).

The absolute size of SLN was significantly different among the weight groups. All the three dimensions were significantly different between ≤ 10 kg and ≥ 30.1 kg categories. The DV dimension was different, also between ≤ 10 kg and 10.1 to 30 kg (Table 4).

**Table 4:** Mean values of the lymph node size and ratios for different weight categories (*n* = 27)

Parameter	≤ 10 kg (n = 09)	10.1 to 30 kg (n = 08)	≥ 30.1 kg (n = 10)
CrCd (mm)	8.9*	13.2	15.0*
DV (mm)	3.5*^#^	6.1^#^	6.2*
LL (mm)	3.6*	4.6	5.5*
DV/CrCd	0.40	0.50	0.45
LL/CrCd	0.48	0.38	0.40
CrCd/St2DV	2.62	2.16	2.90
DV/St2DV	0.53	0.49	0.40
LL/St2DV	0.57*^#^	0.37*	0.36^#^

*St2DV *dorso-ventral dimension of the 2nd sternebra. Numbers labelled with the same sign (* or #) are significantly different (*p* < 0.05). Numbers without any sign are not significantly different.

From the ratio values, only LL/St2DV parameter was significantly different between ≤ 10 kg and 10.1 to 30 kg (*p* = 0.03) and between ≤ 10 kg and ≥ 30.1 kg (*p* = 0.01) (Table 4). All the other ratio values were not significantly different between weight groups (*p* > 0.05).

The mean precontrast and postcontrast density of the lymph node were significantly different (11.7 HU and 59.3 HU, respectively; *p* < 0.05). The precontrast and postcontrast heterogeneity values of lymph nodes were significantly different (12.0 HU and 16.9 HU; *p* < 0.05). The precontrast and postcontrast density of the perinodal fat was not significantly different (-104.6 HU and − 102.0 HU; *p* = 0.178). Descriptive statistics for density and heterogeneity values of SLN and density values of the perinodal fat are presented in Tables 5 and 6, respectively.

**Table 5:** Descriptive statistics for the density values of sternal lymph nodes (*n* = 27)

Statistical variable	Precontrast density (HU)	Postcontrast density (HU)	Precontrast heterogeneity (HU)	Postcontrast heterogeneity (HU)
Mean ± SD	11.7 ± 15.83	59.3 ± 29.67	12.0 ± 5.39	16.9 ± 6.39
95% CI for the mean	5.5 to 18.0	47.6 to 71.0	9.9 to 14.2	14.4 to 19.5
Minimum	-36.6	-5.8	4.2	5.4
Median	16.5	62.5	11.4	17.4
Maximum	39.1	119.7	27.8	35.9

**Table 6:** Descriptive statistics for the density values of perinodal fat (*n* = 27)

Statistical variable	Precontrast density (HU)	Postcontrast density (HU)
Mean ± SD	-104.6 ± 19.4	-102.0 ± 17.9
95% CI for the mean	-112.3 to -96.9	-109.1 to -94.9
Minimum	-138.5	-132.8
Median	-104.2	-100.6
Maximum	-33.4	-39.2

## Discussion

This retrospective computed tomography study aimed to characterise SLN in dogs without any identifiable pathology to serve as a rule of thumb for clinical purpose.

One dog was excluded from the study because SLN could not be identified. There are two possible explanations for this observation. Given the young age and small size of the dog, the lymph nodes could be tiny to be seen on CT images or were not developed.[1] In all the other dogs, at least one SLN was identified. Almost all SLN in this study were well defined with smooth margins. Only in one dog, the lymph nodes margins were not sharp, but still identifiable and easy to assess for this study. The SLN margin perception, in this case, could be influenced by the size of the dog and scan parameters.

Sagittal plane images were used for more accurate measurement of the SLN. DV dimension was relative to the SLN (perpendicular to CrCd dimension). Most of the SLN has an oblique orientation in the sagittal plane with a cranial pole of SLN more dorsal than the caudal pole. Therefore, the DV dimension is falsely bigger if measured from the transversal plane images.

We have found a negative correlation between age and the DV dimension of SLN. This association was already published for medial retropharyngeal lymph nodes in dogs[11] and abdominal lymph nodes in cats.[12] No information regarding the correlation between SLN size and age was previously published.[3, 8] The absolute size of SLN in all the three dimensions was positively correlated to the body weight. The same was already published for the medial retropharyngeal lymph nodes in healthy dogs.[11] However, previous studies dealing with SLN measurements found only the DV dimension to be positively correlated to the body weight in healthy dogs.[3, 8].

The CrCd dimension was the largest one, and LL was the shortest in all SLN in our study. The LL dimension was almost 2 to 3 times less than CrCd. In previous publications, DV/CrCd ratio is commonly used as the short-to-long axis ratio.[3, 5, 8, 13] However, we calculated two different short-to-long axis ratios in our study, DV/CrCd and LL/CrCd. None of these short-to-long axis ratios was significantly different between the weight groups and could be used as a criterion for lymph node size evaluation among dogs of variable size. None of the absolute-size variables or ratio-size values was significantly different between the medium-sized dogs (10.1 to 30 kg) and the large dogs (≥ 30.1 kg); however, this should be tested in the future on a large group of dogs.

This study showed a significant difference between the precontrast and postcontrast density of SLN. This finding is in concordance with already published results for SLN in healthy dogs.[8] The lymph nodes are highly vascularised structures and therefore, strongly contrast-enhancing.[14] The very low precontrast density of SLN (11.7 HU) could be explained as a result of partial volume artefact. However, we limited the size of the region of interest (ROI) to exclude the very peripheral zone of the lymph node. Still, some of the lymph nodes were very small, and the measured density could be falsely lower because of the perinodal fat and the partial volume artefact in CrCd (z-axis). Another explanation of the low precontrast density could be the intranodal fat; however, in most of the nodes, the fat was not visible in the nodal hilus. The standard deviation for the precontrast density was too high (15.8 HU) and higher than the mean value for the precontrast density (11.7 HU). This means that there was a wide range of measured densities. There were four dogs where the measured mean density was negative, and it has influenced our results. Based on these results, we can say that the precontrast appearance of SLN is heterogeneous, and the postcontrast appearance is homogenous. These conclusions are based on a subjective interpretation of numerical data from a single measurement. However, no objective interpretation of such data exists. The precontrast and postcontrast heterogeneity values were significantly different based on the statistical analysis, but we think that this particular difference is of no practical value.

The previous publication stated normal SLN as homogenous in a precontrast study and did not evaluate this parameter after contrast administration.[3] A more recent publication has measured the heterogeneity of SLN and has stated that the SLN parenchyma was homogenous and no significant difference was found between precontrast and postcontrast heterogeneity values.[8].

The first limitation of the study is the retrospective design and heterogenous CT settings, that could affect all the measured variables. Another limitation is the low number of dogs in the study and a wide range of age and weight. It is a consequence of including only dogs without any pathological conditions that could affect the appearance of SLN. A single observer measurement is also a limitation of this study, and a potential human error could influence the data.

A future study should be aimed at the differences between the medium-sized and large dogs, and also to address the value of intra- and interobserver variability on the size and density measurements.

## Conclusions

Based on our results, the DV dimension of SLN is smaller in older dogs compared to young dogs. We recommend using the short-to-long axis ratios for SLN size evaluation or ratio values of the individual dimension to the DV dimension of the second sternebra. All these ratio values were not significantly different between the weight groups of dogs and could be used when comparing lymph nodes in dogs of different size. The data about the heterogeneity of the lymph nodes are not clear from this study and needs to be thoroughly studied in the future.

## Methods

### Animals

Imaging data from the Small Animal Clinic, University of Veterinary and Pharmaceutical Sciences Brno between December 2012 and October 2017 were retrospectively reviewed. Totally 1407 CT examinations of canine patients were found. From these data, only CT examinations of the thoracic and abdominal cavity or total-body examinations were selected (238). From these examinations, we have selected 28 examinations that were classified as normal on the base of no blood work and urinalysis abnormalities, and CT findings of unrelated SLN diseases. None of these dogs had pleural nor peritoneal effusion, nor any pathology that could potentially affect SLN. Indeed, this group consisted of patients with degenerative disk disease and patients without any obvious abnormality on CT images within the draining area of SLN. Sample size calculation was not appropriate as it was a retrospective study with clearly defined inclusion criteria.

The breed, age, weight and sex were recorded. Dogs were divided into three weight groups; ≤10 kg, 10.1 to 30 kg and ≥ 30.1 kg. Informed client consent was obtained before each CT examination.

### CT examination

All of the CT examinations were performed on a 16-multislice unit (LightSpeed, GE HealthCare, Milwaukee, Wisconsin, USA) with the helical acquisition, automatic mA, 100 or 120 kV, rotation time 0.5—0.7 s, slice thickness 1.25—3.75 mm, pitch 0.938 or 1.375, reconstructed in a soft-tissue algorithm. The DFOV was customised to the size of the patient, and the matrix was 512 × 512. All images were displayed in a soft-tissue window (WW 350, WL 50). All of the dogs received iodine non-ionic contrast medium iomeprol in a dose of 600 mg I/kg (Iomeron 300, Bracco Imaging Deutschland GmbH, Konstanz, Germany). Contrast medium was administered into the cephalic vein with a power injector (MCT Plus, Medrad, Indianola, IA, USA) at a rate of 3 ml/s. All of the postcontrast series used in the study were acquired 90 to 120 s after the contrast medium administration. All of the settings in postcontrast series were the same as for the precontrast examinations. Dogs were scanned in sternal recumbency.

### CT measurements

**Fig. 4:**
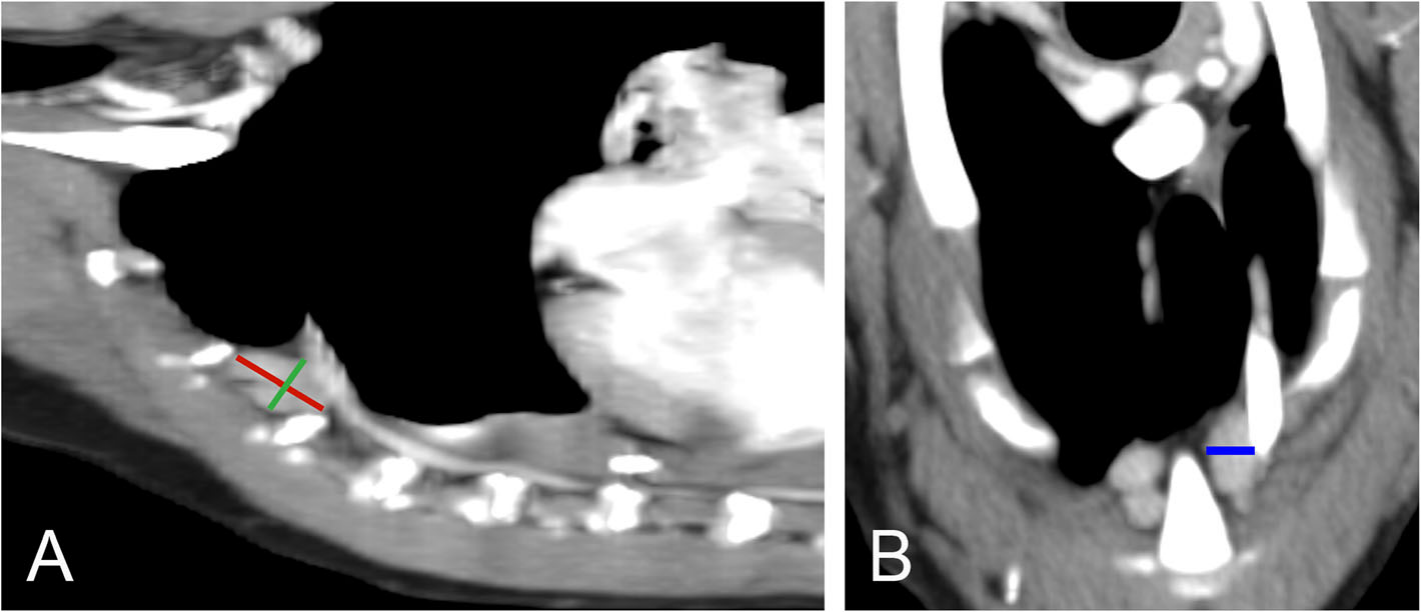
Sagittal and transversal postcontrast CT images show the size measurement of the sternal lymph nodes. Sagittal (**a**) plane postcontrast CT image shows the orientation of the electronic calipers for CrCd (red line) and DV (green line) dimensions. Transversal (**b**) postcontrast CT image shows the orientation of the electronic calliper for LL (blue line) dimension. The right side of the animal is on the left side of the transversal image

**Fig. 5:**
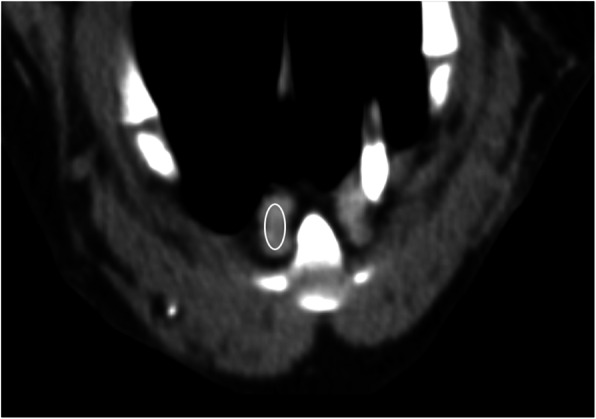
Transversal postcontrast CT image shows the density measurement of the sternal lymph node. Elliptical ROI (white) is drawn over the largest sternal lymph node in transversal CT image. The periphery of the lymph node is not sampled to avoid the partial volume artefact. The right side of the animal is on the left side of the transversal image

Images were reviewed by a single observer (LS) using a DICOM software (TomoCon Workstation version 22, TatraMed Software s.r.o., Bratislava, Slovakia). Multi-planar reconstructions were made to identify SLN. The number, location, size, density and heterogeneity of SLN were recorded in all the dogs. The location of SLN was assessed as a position relative to the adjacent sternebra. The size of SLN was measured in three orthogonal dimensions on transversal and sagittal postcontrast images. The sagittal images were used to measure CrCd and DV dimension (Fig. 4A). The transversal images were used to measure the LL dimension of SLN (Fig. 4B). Two different short-to-long axis ratios were calculated, one ratio was calculated as DV to CrCd dimension, and the other one was LL to CrCd dimension. The DV dimension of the second sternebra (St2DV) was measured from images reformatted in a sagittal plane, and a ratio of all the SLN dimensions to St2DV was calculated to account for differences in body size. All the measurements were recorded in mm. Density and heterogeneity of SLN were measured with a circular/elliptical ROI tool (Fig. 5). The size of ROI was adjusted to encompass the largest possible area of SLN without the very peripheral zone of SLN to avoid the partial volume artefact. Measured mean attenuation value was used for density assessment and measured standard deviation for heterogeneity assessment [15]. All of the measurements were done on the biggest SLN.

### Statistical analyses

A commercial software (Minitab 16, Minitab Inc., Coventry, UK) and Real Statistics Resource Pack software (Release 6.3) for Microsoft Excel (version 16.26) were used for statistical analyses. ANOVA and post-hoc Tukey test were used to compare the SLN size in each dimension among the weight groups. Size of SLN and St2DV were correlated to the weight and age using the Pearson correlation coefficient. Precontrast and postcontrast values of density and heterogeneity were compared using the paired T-test. Statistical significance level was set at 0.05.

## Data Availability

The datasets generated and analysed during the current study are available in the patient archiving and communication system (PACS) at the Small Animal Clinic, Faculty of Veterinary Medicine, University of Veterinary and Pharmaceutical Sciences Brno and are available from the corresponding author on reasonable request.

## Abbreviations

ANOVA: Analysis of variance
CI: Confidence interval
CrCd: Craniocaudal
CT: Computed tomography
DICOM: Digital imaging and communications in medicine
DV: Dorsoventral
HU: Hounsfield units
kV: Kilovolt
LL: Laterolateral
mA: Miliamper
ROI: Region of interest
SD: Standard deviation.
SLN: Sternal lymph node
St2DV: Dorsoventral dimension of the 2nd sternebra
WL: Window level
WW: Window width
